# The Uptake and Impact of a Label for Peer-Reviewed Books

**DOI:** 10.3389/frma.2021.746452

**Published:** 2022-01-04

**Authors:** Eline Vandewalle, Raf Guns, Tim C. E. Engels

**Affiliations:** Faculty of Social Sciences, Centre for Research and Development Monitoring (ECOOM), University of Antwerp, Antwerp, Belgium

**Keywords:** social sciences and humanities, peer review, scholarly books, book publishers, performance-based funding

## Abstract

This article presents an analysis of the uptake of the GPRC label (Guaranteed Peer Reviewed Content label) since its introduction in 2010 until 2019. GPRC is a label for books that have been peer reviewed introduced by the Flemish publishers association. The GPRC label allows locally published scholarly books to be included in the regional database for the Social Sciences and Humanities which is used in the Flemish performance-based research funding system. Ten years after the start of the GPRC label, this is the first systematic analysis of the uptake of the label. We use a mix of qualitative and quantitative methods. Our two main data sources are the Flemish regional database for the Social Sciences and Humanities, which currently includes 2,580 GPRC-labeled publications, and three interviews with experts on the GPRC label. Firstly, we study the importance of the label in the Flemish performance-based research funding system. Secondly, we analyse the label in terms of its possible effect on multilingualism and the local or international orientation of publications. Thirdly, we analyse to what extent the label has been used by the different disciplines. Lastly, we discuss the potential implications of the label for the peer review process among book publishers. We find that the GPRC label is of limited importance to the Flemish performance-based research funding system. However, we also conclude that the label has a specific use for locally oriented book publications and in particular for the discipline Law. Furthermore, by requiring publishers to adhere to a formalized peer review procedure, the label affects the peer review practices of local publishers because not all book publishers were using a formal system of peer review before the introduction of the label and even at those publishers who already practiced peer review, the label may have required the publishers to make these procedures more uniform.

## Introduction

Several countries have adopted performance-based research funding systems (PRFSs) based on bibliometric indicators. At the same time, researchers and policy makers have realized that indicators need to be adapted to the specific nature of scholarly communication in the Social Sciences and Humanities (SSH). The specificities of much of the research in the SSH disciplines have made an all-too straightforward approach toward the counting of publications for evaluative purposes unsuitable for the SSH (Nederhof, 2006). The research output of the SSH is diverse in terms of topics, audiences, publication channels and formats, creating—in the words of Diana Hicks—a “frankly messy set of literature” (Hicks 2005, p. 2). Whether bibliometric indicators are (or can be made) suitable measures for funding allocation for SSH research depends in large part on their ability to adapt to the specific nature of scholarly communication in the SSH. One of the important differences in publication patterns between SSH disciplines and the STEM fields (Science, Technology, Engineering and Mathematics) is the continued importance of book publications to the Social Sciences and in particular the Humanities (see Larivière et al., 2006; Engels et al., 2018). Moreover, SSH are less well-covered in large commercial indexes such as the Web of Science indexes (owned by Clarivate Analytics) or Scopus (owned by Elsevier). According to Giménez-Toledo et al. (2016), “there exists a clear need for comprehensive databases collecting ‘quality’-indicators for books and book publishers” (Giménez-Toledo et al. 2016, p. 2).

In this paper, we draw our attention toward a book label that has been designed to deal with the problem of which books to include in a national database used for the allocation of research funding: the Flemish Guaranteed Peer-Reviewed Content label or GPRC (Verleysen and Engels, 2013). The GPRC label is a tool for identifying peer-reviewed book publications, so that they can be included in the regional Flemish Academic Bibliographic Database for the Social Sciences and Humanities (Vlaams Academisch Bibliografisch Bestand voor de Sociale en Humane Wetenschappen, henceforth VABB). Publications included in the VABB are taken into account for the PRFS in Flanders, which relies in part on bibliometric indicators. The GPRC label can be understood as an attempt to optimize the Flemish PRFS to be more inclusive for the SSH, especially for books published by publishers based in Flanders. The label was created by the Flemish publishers' association (VUV) to enable books by Flemish publishers to be included more easily in the VABB and thus also in the PRFS. The label offers a novel way to include books in bibliometric indicator-based funding allocation systems by focussing on a formalized peer review procedure and by enlisting the publishers of scholarly work to provide information on the peer review processes of their books. The GPRC label can be seen in this context of an increasing awareness of the importance of books to the SSH and the realization that a more comprehensive view of SSH output is necessary, especially for book publications.

We use the concept bibliodiversity (Giménez-Toledo, 2020), to discuss how the diversity of book publications in the SSH is reflected in the choice to implement the GPRC label but also has consequences for the uptake of the label. The term “balanced multilingualism” was coined by Sivertsen (2018) in an argument in favor of a functional balance between publications in English (as the main international language of science) and local languages. The concepts bibliodiversity and balanced multilingualism are explained in more detail further on. The terms “local” and “locally oriented” are used in relation to different aspects of books that give them a local or regional focus. The first aspect being the local language, which in the case of Flanders is Dutch. The second aspect is the geographical location of the publisher, which is Flanders for all GPRC-labeled books. The third is the topic or content of the publications, which can be focused on Flemish or Belgian case studies. The fourth is the intended audience, which can consist of people within the region or country. This aspect is related to both language (books in Dutch) and contents (e.g., books on Belgian Law).

We consider the GPRC label within the broader regional and international context. While it is difficult to make causal claims about the effects of indicators on research practice, we attempt to define the possible social and normative implications of the label as it functions within the Flemish PRFS. We also analyse how the label has been taken up by the different SSH disciplines in Flanders. In this regard, we will focus specifically on two disciplines that have made use of the label frequently: History and Law. We also discuss the possible effects of the label on peer review practices. Previous articles have discussed the creation and potential of the GPRC label (Verleysen and Engels, 2013), and its potential pitfalls (Borghart, 2013).

The rest of the paper is structured as follows. In section ‘Background: The GPRC Label’, we provide an in-depth discussion of the context of the GPRC label and its place within the Flemish PRFS. The specific make-up and context of the label prompts us to reflect on a number of key implications of the GPRC-label. We analyse the uptake of the GPRC label quantitatively in the section ‘Analysis of the Data’. In this section, we also include the insights gathered through in-depth interviews with three experts. Finally, in the last section, we provide a more in depth discussion of the relevance of the GPRC label, with specific attention to the concepts of bibliodiversity and multilingualism.

## Background: The GPRC Label

### Local Context

#### The Flemish PRFS: the BOF Key

The Flemish PRFS is the funding allocation model that forms the backdrop of the GPRC label. Luwel (2021) has recently described the Flemish PRFS in detail. In Flanders, the allocation of funds between universities has shifted from a model based primarily on input indicators (numbers of students) to a model focusing on output indicators. Part of the university funding is specifically directed to the funding of fundamental research: the BOF-key. In 2003, this funding allocation model was extended to consider publication counts and citations as well as input parameters. This has resulted in a “metrics-heavy scheme” (Luwel 2021, p. 2). The allocation model used in Flanders shows some similarities with the models used in the Nordic countries, originally developed for Norway (Engels and Guns, 2018).

The system initially exclusively used the Web of Science (WoS) indexes for the bibliometric part of the indicator. Mounting pressure from academics (especially SSH researchers) coupled with a growing awareness of the limitations of the large citation indexes for assessing research in the SSH prompted the Flemish government to devise a new way for publications to be counted by creating a regional database: the VABB, which lists all peer-reviewed publications by researchers affiliated to an SSH unit at one of the Flemish universities (Engels and Guns, 2018). Inclusion in the VABB is based on the judgement of 18 senior scholars who form the Authoritative Panel (“Gezaghebbende Panel” in Dutch or GP). With the inclusion of the VABB, a comprehensive overview is kept of all peer-reviewed literature published by SSH scholars at Flemish universities. The requirements for inclusion in the database were established by the Flemish government and include, as a formal requirement, that publications have undergone a process of peer review prior to publication. In order to identify which publications can be considered peer-reviewed, different methods exist. Besides publications indexed in WoS – the Social Sciences Citation Index, the Science Citation Index Expanded, the Arts and Humanities Citation Index and the Conference Proceedings Citation Indexes –, which are automatically considered to be peer reviewed, lists of journals that are not indexed in these citation databases are included after judgement by the GP. Similarly, the GP maintains a list of publishers that employ peer review for their books. However, identifying peer-reviewed books on the publisher level complicates the possibility of including “hybrid” publishers of scholarly and non-scholarly books, while the identification of peer review on the level of individual books is a cumbersome task. Individual book publications can also be included in the VABB through an appeals procedure. The GPRC label creates the possibility of including publications without having to go through the appeals procedure. Meanwhile, another way to include books in the VABB is to approve book series instead of individual publications or book publishers. Publishers that use the GPRC label can also have their book series approved for the VABB. However, the inclusion of book series is not limited to the Flemish publishers. In this study, we limited our attention to the discussion of the GPRC label as used for individual publications.

The intention of the Flemish government was to stimulate universities to conduct a strategic research policy and to provide incentives toward the enhancement of research quality and a focus on fundamental research (Bof besluit, 2019). The BOF-key is thus an instrument that allows the Flemish government to provide incentives for the universities when it comes to making strategic decisions. The GPRC label was created in the context of this allocation model as a tool for including peer-reviewed book publications by Flemish publishers more easily in the VABB. For the Flemish publishers' association (VUV), the motivation was to make publishing with Flemish publishers more attractive for researchers at Flemish universities.

#### The Creation of the GPRC Label

As mentioned above, the GPRC label was created to find a way for books published at Flemish book publishers to be included in the VABB. The label is trademarked by the VUV. There are different ways for book publications to be included in the VABB. Even though the GPRC label was announced before the first version of the VABB, the effect of the GPRC label can only be studied now because the VABB uses a 10-year window. The first way for book publications to be included was through a list of approved publishers, which included predominantly international scholarly publishers who exclusively published peer-reviewed books. For local publishers with a mixed or hybrid portfolio of both peer-reviewed and non-peer-reviewed publications, this meant that they would never be able to appear on the list and thus never have their book publications be included in the VABB without going through the appeals procedure. The VUV feared that scholars at Flemish universities would target their publications toward (almost exclusively international) publishing houses that did appear on the list, thus creating a disadvantage for local publishers. Moreover, the situation created an inequality between the two Flemish publishers already on the list (Brepols and Peeters) and other Flemish publishers not on the list. The system could also mean a disadvantage for disciplines that publish more locally oriented books, such as Law or History. Furthermore, the inclusion of local publishers would contribute toward making the VABB database more comprehensive.

The GPRC label offers a way for books to be included in the VABB on an individual basis. The procedure for the GPRC label is as follows. The publisher holds on to a peer review dossier that includes at least the following parts:

(1) The table of contents of the publication
(2) The affiliation of the reviewers
(3) A chronological overview of the peer review process
(4) A minimum of two reviews
(5) A formal confirmation that the reviewer authorizes the publication with the label.

The ultimate decision on inclusion in the VABB remains the responsibility of the GP. The publisher has to submit the peer review dossier to the GP when requested. The panel does not review all publications with the label, but a selection each year from a number of publishers. The panel does not read the actual reviews submitted by the reviewers, their judgement of the peer review dossier is based solely on the formal criteria. The procedure for the inclusion of book series is similar. Publishers still have to hold on to a peer review dossier, which can be requested by the GP.

#### Examples of Labels for Peer-Reviewed Books

In their discussion of the GPRC label, Verleysen and Engels (2013) already discussed the international opportunities of a label for peer-reviewed books. An international label has not yet been created, but so far, a label modeled on the Flemish GPRC label has been adopted by two other countries. In 2014, the Federation of Finnish Learned Societies -introduced a label for peer-reviewed books and articles. The Finnish label is meant to “inform peer review practices used in Finnish scholarly publishing with the best and ethically sound international standards,” and to “make it easier for students, researchers, libraries, administrative actors or other users of research literature to recognize peer-reviewed publications” (Label for Peer-Reviewed Scholarly Publications, 2015). The Federation of Finnish Learned Societies requires publishers to describe their peer review procedure on their website and to store the documentation related to the peer review process. The requirements of the peer review process are similar to those of the GPRC, i.e., a minimum of two independent reviews is required.

A quality label for scholarly book publications has also been established in Sweden: Kriterium, a label that was established in a bottom-up way (Hammarfelt et al., 2021). The Kriterium board manages the peer review process itself, appointing an academic coordinator, who in turn picks two reviewers and oversees the review process. The ultimate decision on acceptance or withdrawal lies with the Kriterium board. This is different from the GPRC-label, where the peer review process is managed by the publisher and the GP only checks whether the formal aspects of peer review have been satisfied. Another interesting feature of the Kriterium label is that all publications have to be made accessible open access on Kriterium's website.

Meanwhile, Central and Eastern European countries know a tradition of publishing scholarly books with the names of the reviewers. Using data from Poland, Kulczycki et al. (2019) characterized this practice as an open-identity peer review label that could be used to delineate between peer-reviewed and non-peer-reviewed publications.

### Implications of the GPRC Label

#### Peer Review

The use of the GPRC label may have social as well as normative implications. Firstly, as mentioned before, the label requires books to have undergone a peer review procedure, thus further institutionalizing peer review as a delineating criterion for what is seen as scholarly and what should count toward the PRFS. Whereas peer review has been the gold standard of quality in (international) journal literature for many decades, peer review has historically not been the primary way to ensure the scholarly value of book publications. Scholarly books have often been published by university presses who upheld the quality through the editorial processes of choosing authors and topics carefully and guarding the scholarly nature of their publications. Pochoda (2013) mentions in this context the “stable, bounded, continuous, well-ordered and well-policed” analog scholarly publishing system that knew its heyday in the mid-20th century. Pochoda (2013) also mentions that the subjection of manuscripts to external review only dates back to the 1960s. Before that time, book reviews and informal barriers were used to ensure quality. Book reviews are still being written and read by scholars (Hartley, 2006), and are a way for peers to make a decision about whether or not to invest time in reading a particular scholarly book. Meanwhile, the peer review system is inextricably linked with the rise of the academic journal with double-blind peer review being introduced in the mid-20th century (Gould, 2012).

The pressure on academics to exclusively publish peer-reviewed works is increasing, and this can potentially affect publication practices in SSH. As already mentioned, researchers in the SSH publish a variety of works, including peer-reviewed scholarly articles, but also books directed at a wider audience. Hicks (2005) argues that scholars in the social sciences and humanities contribute more to the so-called “national literatures.” Meanwhile, scholars in the humanities have often complained of the narrow focus of research evaluations on internationally oriented journals which affects the day-to-day practice of scholars (see e.g., De Wever, 2007).

Today, external review (or peer review) is the norm for academic journals, as well as for many scholarly book publishers. The GPRC label is an attempt to include all peer-reviewed literature written by local scholars, not only the publications indexed in the major citation databases or published by publishing houses with an international reputation. In this way, the label can help scholars to continue to have a diverse portfolio of articles in international journals indexed in the major indexes as well as locally relevant publications in journals and books, often in Dutch, provided that they have been subjected to peer review. However, we have to remember that the GPRC label was introduced in a local (Flemish) context, and was also made available to publishers that do not have an exclusively academic/scholarly portfolio. Even though peer review has become the standard quality criterion of scholarly work, it is not necessarily performed systematically at local publishers. Some of the publishers who now publish GPRC books, previously may not have performed peer review of scholarly books in a systematic way. As the GPRC is an element within a PRFS that recognizes peer review as the main delineating factor between scholarly and not scholarly, it may result in some disciplines using peer review for a larger portion of their publications and it may tempt Flemish publishers toward using peer review and standardizing their peer review procedures.

Another important element to take into consideration is how peer review is being defined. A recent study by Giménez-Toledo et al. (2017) looks at how peer review is defined in the context of different PRFSs in European countries. Giménez-Toledo et al. (2017) state: “There is a diversity of approaches to defining peer review and applying it in the evaluation process: from the specific definitions and requirements in the case of the Finnish and Flemish labels to the use of existing information on scholarly publisher's peer review practices by evaluation agencies in the case of Spain.” Moreover, Pölönen et al. (2020, p. 3) point out that identifying peer-reviewed publications is ambiguous, and that within PRFSs, peer review is typically defined technically, “focussing on the existence of a recognizable pre-publication procedure.” In the case of GPRC, the focus on the existence of a formal peer review procedure relates to the requirements for inclusion in the evaluation system as put forward by the Flemish government: the BOF-decree regulations.

#### Bibliodiversity and Multilingualism

The concept *bibliodiversity* was introduced by Giménez-Toledo (2020) to stress the importance of taking into account a variety of publications from the full range of small and medium-sized regional publishers to the large international publishing conglomerates, but also to emphasize the diversity of topics, languages, perspectives and methodologies in scholarly publishing. In the Social Sciences and Humanities not only different publication types are used, but also a mix of books in local languages and English, topics of local relevance, books directed at different audiences which creates a diverse publication field.

Giménez-Toledo (2020, p. 3) points out that “lack of insight implies practically that there is going to be an adequate recognition for those books published by the large publishing companies with an international profile but not for the more national oriented and smallest ones.” The GPRC label caters specifically to this point of having a balanced PRFS where the evaluation does not disfavor smaller and medium-sized national publishers.

Protecting this diversity is a complex problem as the scholarly book publishing market is influenced by internal developments of scientific fields, the financial resources of universities and university libraries and the incentives provided by the evaluation systems and PRFSs.

We can identify some changes in the scholarly book publishing market, influenced by these various elements. Where the scholarly book publishing market used to be dominated by university presses, there now exists an increasing concentration among big conglomerates (Pochoda, 2013). In a study about the Flemish publishing landscape, Guns (2018) found that there was a relatively high concentration of books among a few publishing houses. The concentration was larger for peer-reviewed books, suggesting that researchers publish their scholarly books at a few important publishers. The increasing concentration of publishers has played an even bigger role for journal publishers (Larivière et al., 2015).

A decline of university presses and an ascent of commercial publishing houses likely has consequences for the content of books as well. Moreover, large players, such as Oxford University Press and Routledge have started to dominate the market. Mañana-Rodríguez and Giménez-Toledo (2018) found that university presses tend to be more multidisciplinary, as their mission is to some extent different from commercial publishing houses. However, some university presses have become privatized, making the distinction less clear.

Not only the size of publishers, but also the location of publishers can be an element in bibliodiversity. In a study by Verleysen and Engels (2014), the internationalization of scholarly book publishing was studied for the Flemish SSH. Both the evolution of the internationalization and the different levels of internationalization between SSH disciplines were analyzed. The analysis showed that for the Social Sciences, publishers are located more toward the U.K. The publishers of publications in the Humanities, however, veer more toward Flanders and continental Europe.

The bibliodiversity also concerns diversity in terms of language. The general trend in scholarly publishing is that researchers tend to write more in English, as it is an international language of science, although there are important regional differences as well as differences between fields of science. Comparing the language of SSH articles in seven national databases, Kulczycki et al. (2020) found that in the Nordic and Western European countries, including Flanders, the dominant language for publications is English, while this is not the case for Central and East-European countries. Moreover, they report the highest shares of local language use in Law, History and Archaeology, and Arts. Multilingualism is therefore especially important to the SSH which contain fields of study where publishing in languages other than English is more common. It is also relevant in particular to non-English speaking areas.

The use of English as an international language of science has been widely debated (Tardy, 2004; Stockemer and Wigginton, 2019). While one of the foci of this debate is the disadvantage faced by non-native English speakers, especially in developing countries, when disseminating their research results in international elite journals (Salager-Meyer, 2014), another focus of the debate surrounding English as a language of science has been the importance of native languages to the communication of research in local contexts. For example, Sivertsen (2018) has argued that “to fulfill its responsibilities, science needs to be multilingual.” In this context, Sivertsen (2018) has coined the term “balanced multilingualism,” where some publications are written in local languages and thus appreciated by the professionals or the general public while others are disseminated to international peers in English-language journals.

Multilingualism is important because the language in which a scholarly work is written has an effect on the audience it can have. A scholarly book written in Dutch will not be read by international peers who do not understand Dutch. On the other hand, a scholarly book written in English will be less likely to attract local readers, lay people or professionals to read the book. The GPRC label, as a local label, may play a role in the multilingualism of SSH publications in Flanders. While previous studies have warned about the increasing homogenization of publications as a possible adverse effect of current research evaluation systems (Dahler-Larsen, 2018), the GPRC label provides the opportunity for local researchers to have their Dutch-language publications included in the PRFS.

We thus use the concept bibliodiversity to denote the diversity of publication channels and types and we use the concept balanced multilingualism to argue that rather than a complete dominance of English or a return to local languages to communicate research, a functional balance needs to be found between the use of English as the international language of science and local languages to communicate research findings, specifically with local subjects. For the SSH, a research funding mechanism based on bibliometric indicators should take into account the specificities of SSH research, with particular attention toward bibliodiversity and balanced multilingualism.

#### The Trickling Down of Incentives

The GPRC label can simplify the inclusion of book publications in the PRFS, which can affect the allocation of funds. Consequently, it is possible that this advantage at the institutional level translates into incentives at the lower levels, where departments can show that their output contributes to the funds received by the institution. However, as the GPRC label is only relevant to a small part of SSH financing and other ways to include peer-reviewed book publications exist, the potential financial benefits of the use of the label are limited. Moreover, researchers face different kinds of pressures, including pressures to publish in influential international journals which may offset the relatively small incentives for publishing GPRC-labeled books. In order to understand how the GPRC label fits within the larger PRFS and the Flemish context, several aspects regarding the trickling down of incentives from the PRFS to actual researcher practice should be taken into consideration.

Firstly, even though PRFSs are meant to incentivize certain changes in institutions, e.g., increasing the publication output, the incentive structures are not always straightforward: “incentives in evaluation systems do not only reinforce each other, but may also work in opposite directions” (Hammarfelt and de Rijcke 2015, p. 74). While the GPRC label seems to offer an incentive toward publishing books at regional Flemish publishers, it is only a small part of the PRFS. The BOF-key as a whole incentivizes publishing in different kinds of peer-reviewed publication channels, although the inclusion of citation-related parameters may put stronger emphasis on WoS-indexed journals. The GPRC label is meant to reduce the possible disadvantage that could result from an over-emphasis on international publishers and too little attention to national literatures and scholarly books.

Secondly, the mere existence of metrics, measures and evaluation systems can influence managers' and researchers' decisions. Researchers may be impacted for example because “academics perceive the expectations built into (research evaluation systems) and interpret them as signals of what society values about their research” (Gläser and Laudel 2007a, p. 132). This effect of evaluation systems operates regardless of financial incentives.

Besides incentives within the PRFS, there are also external incentives. Publications in international journals, preferably with high impact factors, are generally considered to be more prestigious and are more important to researchers' careers. Researchers can engage in what Gläser and Laudel (2007b) have called “amateur bibliometrics.” Therefore, the potential effects of GPRC label should be seen within the whole context of incentives provided by the BOF-key, but also external incentives such as the prestige associated with publishing in internationally recognized publication channels, or the existence of international rankings and metrics. These external pressures could reduce the importance of the GPRC label.

Aagaard uses the concepts *tight coupling, loose coupling* and *decoupling* to explain the different ways in which incentives of a bibliometric indicator trickle down to the individual level (Aagaard, 2015). Aagaard points out an apparent paradox in the Norwegian PRFS. Based on the facts that the financial incentives are not strong, the system is not meant to be adapted on individual levels, the higher education institutions have a lot of autonomy, and the system itself is contested within the sector, a loose coupling or decoupling would be expected, whereby the PRFS would have only very small or no effects on local management practices (Aagaard 2015, p. 728). However, a number of indirect mechanisms and factors may create a mechanism whereby incentives trickle down and external pressures are internalized. Aagaard uses the concept *allure* to denote the temptation for managers to adopt measures they acknowledge to be poor at individual levels, because of the simplification the quantitative measure entails and the seemingly objective nature of the measure. Aagaard proposes the concept *anxiety* to address the uncertainty and anxiety faced by individual researchers about the future importance of the indicator even if the current use is limited or downplayed by managers and administrators. Researchers may anticipate that the indicator may become more important in the future (Aagaard, 2015).

These studies point toward a complexity of mechanisms which makes it difficult to immediately assess the potential effects of a PRFS, or in this case, one element within a PRFS. While the existence of a label such as the GPRC label may affect everyday research, it is difficult to predict to what extent and in what way.

## Analysis of the Data

### Data and Methods

This study uses both qualitative and quantitative methods within a sequential explanatory strategy whereby the focus lies on the quantitative analysis of the data (Creswell, 2014). The qualitative part of the study in the form of in-depth interviews helps to contextualize some of the findings from the quantitative part of the study.

The data used for the quantitative part of the study are publication metadata from the VABB database from the years 2010 (the year of the first GPRC-labeled publications) until 2019. These data are part of the most recent version of the VABB, version 11 (Aspeslagh et al., 2021). The dataset available online only contains the approved publications, not the publications which were not approved for inclusion in the VABB. Thus far, 2,580 publications with the label have been included in the VABB database, compared to 82,403 publications in VABB 11 in total (3%). The metadata in the dataset used in this study includes the publication year, the type of publication, the judgement of the Authoritative Panel (GP), the titles and the language of publication.

The analysis of the VABB data contained descriptive statistics that show the evolution of the number of publications as well as the breakdown of these publications between disciplines. Based on the empirical results from these descriptive statistics, two disciplines (History and Law) were chosen to include in a further analysis of the content. We conducted a categorization of publication titles in terms of local (Belgian/Flemish) or international orientation for these GPRC publications.

For the qualitative part of the study, three in-depth interviews with academics who had come into contact with the GPRC label were conducted. The interviewees were selected because of their experience with the GPRC label as author, member of the GP and/or through their experience in research management. Not all Flemish SSH researchers are familiar with the GPRC label or have a deep understanding of what the GPRC label entails. We decided to interview in particular someone from the discipline Law because the data pointed toward Law as the discipline that made the most use of the GPRC discipline, both in relative and in absolute terms. The in-depth interviews focused both on the respondent's general experience with the GPRC label (as author or policy maker) and some of the empirical results from the first part of the study. The interviews were recorded and transcribed. An ethical clearance was obtained from the University of Antwerp's Ethics Committee for the Social Sciences and Humanities. To safeguard the anonymity of the interviewees, we are not mentioning the specific professional positions of the interviewees.

We identify a few key points of interest for the analysis of the data. Firstly, we want to find out to what extent the label has been used in the past 10 years and whether it has contributed to a more comprehensive inclusion of book publications in the VABB. Secondly, we analyse the languages represented among the GPRC book publications. We analyse to what extent the GPRC label contributes to a “balanced multilingualism.” Dutch language publications are also expected to discuss local topics. Thirdly, we expect the label to be taken up differently by the different SSH disciplines. While some SSH disciplines gear more toward the publication practices of STEM fields, publishing mostly English-language articles in international journals, others have a substantial share of local language articles and book publications, this is especially true for Humanities disciplines.

### The Uptake of the Label and Its Effect on the Inclusion of Books

In this first part, we analyse the evolution of the number of GPRC publications. VABB distinguishes between five publication types, four of which are relevant in the context of GPRC (chapters in edited volumes, edited volumes, monographs, and proceedings papers). We use the term “book publications” to refer to individual publications, which can be book chapters, edited volumes, monographs and proceedings papers.

**Figure 1:**
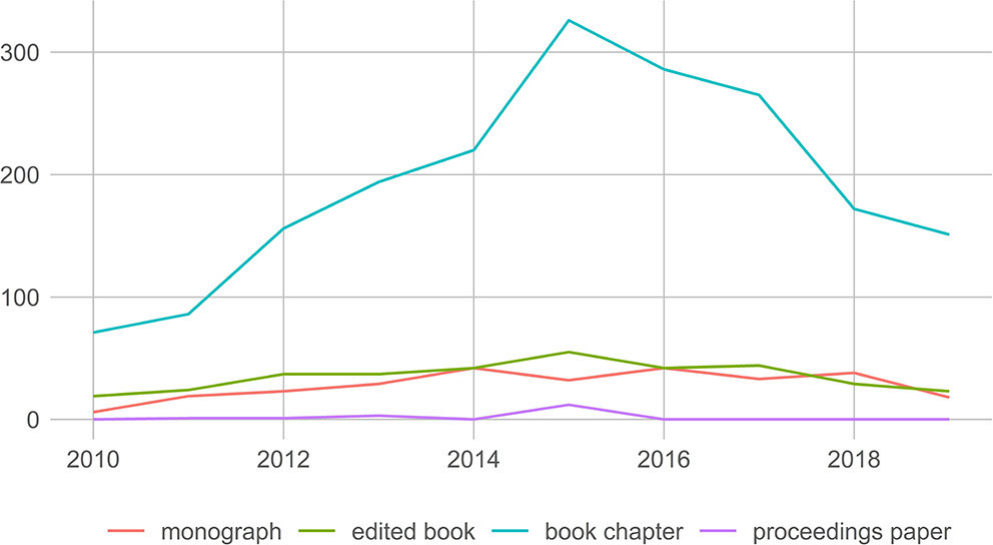
Evolution of the number of publications with the GPRC label, by publication type.

As can be seen in Figure 1, the number of publications written or edited by scholars at SSH departments of Flemish universities that have been accorded the GPRC label increased until 2015 and dropped since then. Caution is necessary in interpreting this decline, since the numbers are reported on the level of individual publications as reported to the VABB. As a result, a few edited volumes with many chapters may have a substantial effect on the numbers. However, looking at individual books (ISBNs) a similar pattern of decline in recent years emerges with a drop since 2016.

The most recent information on how many GPRC books were reported by publishers for 2020 shows a more nuanced picture. The number of books submitted for the last year (books that are not yet included in the VABB and thus do not show on the graph) is 104, almost 30 more than the 77 books that were reported by publishers in 2019. It seems too early to speak of a permanent drop in the use of the GPRC label.

In 2009, the year before the introduction of the GPRC label, 22.3% of book publications submitted by universities (chapters, edited volumes, and monographs) were included in the VABB. In 2019, the last year for which we have final data, this figure was 43.1%. This means that the inclusion rate for book publications in the VABB has almost doubled in the past 10 years. We hypothesize that this is due to an increase in comprehensiveness of the database (publishers, series and books with peer review are more recognized as such) as well as a stronger inclination to publish peer-reviewed books at the researcher level. The alternative explanation—that universities no longer submit publications they assume will not end up in the database—is unlikely as sorting the publications would be much more time-consuming for them and uncertainty over outcomes makes the responsible people opt for the safer option of submitting everything that might potentially be included. It is, however, possible that publications that do not fit neatly in any of the five publication types are not always submitted.

**Figure 2:**
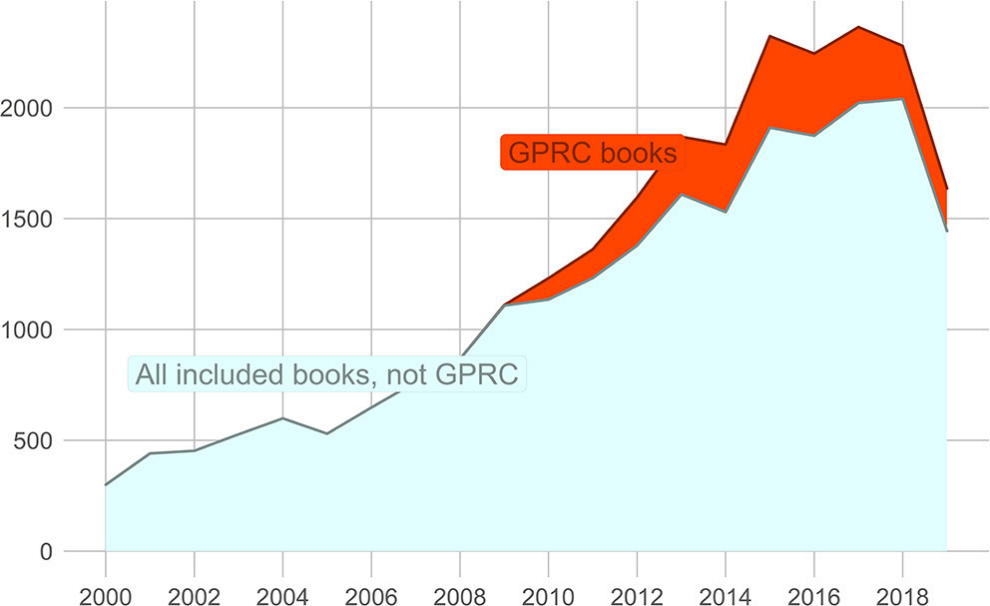
Evolution of the number of peer-reviewed book publications in the VABB.

As can be seen in Figure 2, the number of book publications in the VABB increased roughly linearly. This is in part due to an overall rise in production. As mentioned before, the inclusion of book publications in the VABB takes different forms, with GPRC accounting for a substantial proportion of book publications in the VABB. The highest share of GPRC-labeled publications among VABB book publications was reached in 2015, with 18.3% of book publications included in the VABB. The share dropped to 11.7% in 2019. The increase in the number of book publications can thus not be solely attributed to the GPRC label. However, as the goal is to include all peer-reviewed publications, the GPRC label offers a new way for peer-reviewed publications to be added to the VABB that otherwise would be more difficult to include. It has added to the total of book publications in the VABB, but it is not the sole driving force behind the growth in peer-reviewed book publications.

### Local Orientation of GPRC Publications

In this section, we discuss two aspects concerning the local orientation of publications. The first is language and the second is the content of publications, with topics of local relevance.

As expected, the GPRC label shows a higher proportion of publications in Dutch (the local language) when compared to the language of other publications in the VABB. For the total database, 78.4% of included publications are written in English, with only 16.3% in Dutch. The third language is French (2.89%), the other major national language of Belgium. Among book publications, 73.5% are written in English, and only 16.1% in Dutch. A relatively large proportion of book publications are written in French, 5.8%. English is especially prevalent among WoS-indexed publications, where 96.5% of publications are written in English. For book publications with the GPRC label, only 28.8% of book publications are written in English while the majority, 68.1%, are published in Dutch. 2.5% of GPRC publications are written in French. In this way, the label caters mostly toward local language publications and adds an avenue for Dutch language publications to be included in the VABB. Thus, the GPRC label contributes to a balanced multilingualism, whereby English-language publications in international journals exist alongside a national literature of local language publications, including book publications. With the VABB system, both types of publications are counted toward the PRFS, provided they have been subjected to peer review prior to publication.

Another aspect to take into consideration, is the content of publications. While it was not possible to study all GPRC publications in detail, an analysis of the list of GPRC titles taught us that several publications in the field of Law concern expositions of parts of the Belgian system of law. We conducted a categorization of the titles of 224 publications in Law that were either classified as edited book publications or monographs. We assigned publications to the category of Belgian or local issues if they contained explicit references to the locality (e.g., if they contained the word “Belgium”) or if they could reasonably be assumed to deal with a local topic (e.g., “Handboek algemeen huurrecht” – “Handbook for general tenancy law” applies to the local tenancy law). Publications were assigned to the category of EU or European Law if their titles referenced either the EU or Europe. Most publications that were categorized as dealing with local topics did not use a toponym in the title, probably because the fact that they discuss Belgian law was assumed to be self-evident. Of the 224 publication titles we analyzed, 110 publications were found to concern Belgian or local law or criminology subjects. Twenty-four publications were found to concern EU or European law. Our approach is probably an underestimation, since it was based on a “common sense” categorization, where only publications that were very clearly local in nature were assigned to the local category. While this is only a rough estimate, it gives an indication of the importance of local topics to GPRC publications in the field of Law and a general impression of which kinds of publications the GPRC label is being used for by Law researchers. As these types of publications benefit in particular the local law practitioners and students, it makes sense for them to be written in Dutch. Eleven of these publications contain the word “handbook,” which can be translated into English as “handbook.” While the Dutch Law publications tend to focus on local law, the English-language Law publications often cover EU law.

Closer scrutiny was also given to publications in the field of History, because book publications have been singled out as important parts of the research output of historians (Verleysen and Engels, 2012). From 113 publications from the field of History, it was immediately apparent that a large portion of them had a focus on Belgium, the Low Countries (current Belgium and the Netherlands) or specific places of the region. Using the same method as for the Law publications or categorizing the historical publications, 39 publications were found to be explicitly referencing either Belgium, a region within Belgium, or the Low Countries.

As we have shown, the GPRC publications are mostly locally oriented. The main reasons for this are that only local publishers can use the label and that book publications in particular are used by different disciplines for local audiences, not only academic peers. For a discipline such as Law, this can be professionals in the field as well as students. For a discipline such as history, this can be an interested lay community.

### The Distribution Among Disciplines

In this part, we analyse how the label has been taken up by the different disciplines. Table 1 shows the number of publications with the GPRC label for the different disciplines. The classification used here is a cognitive classification (Guns et al., 2018), based on the Fields of Science classification. The table includes all disciplines with 15 or more GPRC-labeled publications. This results in the exclusion of non-SSH disciplines. Note that in the FoS classification, the discipline Law encompasses both the field of Law and the field of Criminology. It should also be noted that this classification does not take into account multidisciplinarity. Publications to which more than one discipline was assigned are counted fully for each of the disciplines.

**Table 1:** Overview of the distribution among disciplines.

**Discipline**	**# GPRC-labeled publications**	**# Book publications**	**Total # publications**	**# GPRC/# Book publications**	**# Book publications/total** **# publications**
**Social sciences**					
Law	968	3,394	11,614	28.5%	29.3 %
Sociology	314	2,352	5,988	13.3%	39.5%
Political science	220	1,290	3,983	17.1%	32.6%
Social sciences general	135	294	722	45.9%	44.8%
Economics and business	78	1,693	7,924	4.6%	21.9%
Other social sciences	56	418	1,543	13.4%	27.6%
Psychology and cognitive sciences	51	372	7,030	13.7%	5.4%
Educational sciences	40	638	3,411	6.3%	19.3%
Media and communications	29	145	1,885	20%	8.1%
Social and economic geography	15	104	2,821	14.4%	3.7%
Total social sciences	1,906	10,700	46,921	17.8%	22.8%
**Humanities**					
Arts (arts, history of arts, performing arts, music)	147	893	2,352	16.5%	38%
History	114	1,385	4,673	8.2%	29.6%
Philosophy and ethics	107	1,067	2,904	10%	36.7%
Religion	56	1,583	3,104	3.5%	51%
Languages and literature	222	3,165	8,655	7.01%	36.56%
Total humanities	646	8,093	21,688	7.98%	37.3%

The discipline that has used the label the most is Law. The predominance of Law publications among total publications is not surprising. Both Law and Criminology are disciplines that publish a large amount of publications in Dutch (53% in the last 10 years). Moreover, many Law publications are book publications (31%, compared to 22.7% for the total of VABB). However, the predominance of Law publications among GPRC publications is also related to the nature of scholarship in Law. In many countries, a debate has emerged in recent years about the nature and methodology of research in Law, and the position of so-called doctrinal research (Stolker, 2003; Van Gestel and Micklitz, 2014; Kaltenbrunner and De Rijcke, 2017). This debate also has consequences for PRFSs, as they embody decisions on which publications should be counted.

**Figure 3:**
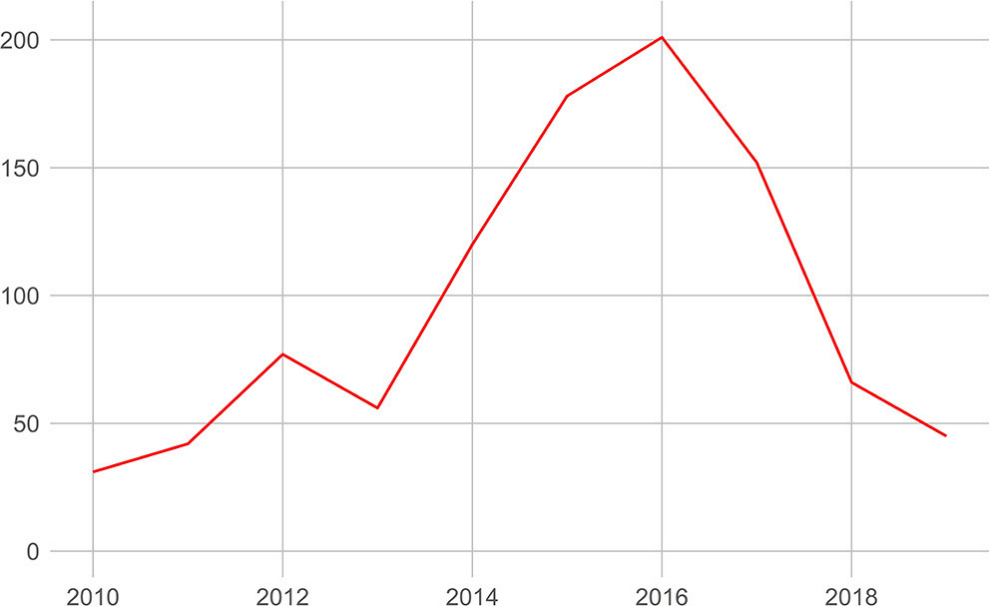
The evolution of the number of GPRC publications in the discipline Law.

Because the discipline Law seems to use the label most often, Figure 3 shows how many law publications were published each year with the GPRC label. Even though the total number of law publications is still modest, which can produce big jumps in the numbers, a clear pattern emerges whereby the number of publications before 2016 rises significantly, to decline quite significantly after 2016.

A second noticeable trend in the distribution between disciplines is that some disciplines use the GPRC label for a larger portion of their book publications. Disciplines such as Law, Political Science and Arts use the GPRC label for a relatively large portion of their book publications (Table 1). Meanwhile, Languages and Literature and Religion, make use of the label for a relatively small portion of their book publications even though they are book-intensive fields. A difference here could be that those disciplines target non-Flemish book publishers or publishers that are automatically included in the VABB. The last column shows the proportion of book publications among VABB publications for each of the disciplines. These levels vary significantly. The highest proportion of book publications can be found for the discipline Religion, where more than half of publications included in the VABB are book publications. Conversely, for a discipline such as psychology, book publications account for only little more than 5% of publications in the VABB.

We now zoom in on the differences between the social sciences and the humanities in terms of how the GPRC label has been taken up. Because the humanities are typically seen as more book-oriented disciplines, we expect the GPRC label to be more relevant to the humanities. We disregard the discipline Law. Firstly because Law has an overwhelming presence in the data and secondly because Law is sometimes also classified as a humanities discipline (e.g., within the classification system used in the VABB which is based on the affiliation of researchers). On the whole, the intuition that a larger proportion of publications from the Humanities would be book publications than for Social Sciences disciplines is confirmed for the VABB data. Thirty-seven percent of publications in the Humanities are book publications, compared to only 23% for social sciences publications. This is not unexpected and in line with previous studies on book publications in the SSH (Verleysen, 2016). More unexpected is that the percentage of GPRC publications among book publications is higher for the social sciences than the humanities. One possible explanation could be that, since book publications are more important to the humanities, researchers were already publishing at international publishers or local publishers with a strong tradition of peer review to reach the international scholarly community. We point here again to the discipline Religion (or Theology), where publication channels that are automatically included in the VABB continue to be used. It is to be expected that scholars make decisions on where to publish, locally and internationally, based on the reputation of the publisher in the field. Strong book publishing traditions in the humanities may result in their most important publishers already being on the VABB list. Some caution should be used in the interpretation of these results. As the GPRC label concerns such a small proportion of total publications, a few publications could alter the picture. Moreover, the distinction between the social sciences and humanities can be a meaningful one, but there also exist large differences in the uptake of the label within each field. For example, for social sciences, the discipline Economics & business does not seem to make much use of the label.

### The Publishers

This last section of the analysis of the VABB data concerns the publishers. Part of the explanation for why some disciplines have made use of the label more frequently than others, lies with the publishers. Many publishers have a clear subject specialization. The Flemish publisher Peeters, for instance, focuses on religious studies, among other topics. Because Peeters is one of the two Flemish publishers whose publications are automatically included in the VABB, their books do not have a GPRC label. This explains why the discipline Religion uses the GPRC label comparatively little.

Table 2 shows the top 5 publishers of GPRC publications. The publisher Intersentia (which belongs to the same parent company as Larcier since 2018) publishes mainly publications in Law. Because of the predominance of Law publications among GPRC-labeled book publications, it appears relatively high on the list. The largest publisher of GPRC-labeled book publications is Leuven University Press, the university press of the largest university of Flanders. University presses of other Flemish Universities—specifically Antwerp University Press and VUB Press, both imprints of ASP (Academic and Scientific Publishers)—also publish books with the GPRC label.

**Table 2:** Top five publishers of GPRC publications.

**Publisher**	**# publications**
Leuven University Press	413
Acco	395
Intersentia	374
Maklu	339
die Keure/La Charte	319

### The Interviews

The three in-depth interviews with experts gave some additional insights into how the label was taken up. We have identified a few key points of interest. The case of Law was discussed more in depth in the interview with a researcher from Law. All three of the experts highlighted some advantages and disadvantages to the GPRC label and spoke from their personal experience in a management position and/or as author of GPRC-labeled books.

One of the implications of the GPRC label we identified earlier on in the study is that peer review procedures have been introduced at publishers who were not used to follow peer review procedures before. One respondent who was more directly involved with the evaluation of peer review dossiers at the GP commented that in the early days of the GPRC label, review dossiers often did not comply with the formal requirements, e.g., the author names or publication title would be missing. The publishers needed some time to get used to holding on to a peer review dossier that complied with the formal criteria. However, the GP does not evaluate the reviews themselves, only whether the reviews are present in the dossiers. For an evaluation of the strength of the actual reviews in the peer review dossiers and whether there has been an evolution in this, a review of the dossiers should be undertaken. This was done for 24 books with the Kriterium label by Hammarfelt et al. (2021).

A few potential flaws of the GPRC label were identified during the interviews. With regard to the peer review process, one of the respondents raised the issue that sometimes the peer review process would be started after the manuscript was finished, and would not induce changes to the book. This undermines the idea of the label as a quality criterium.

A second problem that was identified with regard to the GPRC label is the reluctance by publishers to use it, which could be related to the difficulty of finding reviewers for GPRC books. The empirical results of the study seem to point toward a certain stagnation or even decline in the numbers of GPRC-labeled books. Among the reasons for this, a possible ceiling to the number of books fitting within the GPRC label was mentioned. However, one of the respondents argued that publishers are not eager to have their books GPRC-labeled because they find that it requires too much effort without getting much in return. Speaking from the position of reviewer, a respondent indicated that they also found it too onerous to review a potential GPRC book, on top of the many requests to review journal articles. Another respondent commented on the language barrier making it more difficult to find reviewers for GPRC books as Dutch language books have a smaller potential pool of reviewers. The difficulty of finding reviewers could also be a reason for why publishers may find it cumbersome to go through the procedure for a GPRC label.

With regard to the effect the GPRC label may have on the publication practices of researchers, our respondents indicated that the primary use for the GPRC label is for the researchers to have their books more easily included in the VABB, which is good for their career because VABB publications are recognized within the Flemish system. The interviewees all commented that international publications are important to researchers, and the GPRC label is interesting for books that would have been published locally anyway.

Another possible use for the GPRC- as a label marking *quality* – does not seem to play a role. Researchers do not look specifically for the GPRC label when they are looking through scholarly works. The label is also not internationally recognized. One respondent argued that this is one of the reasons why the label is less interesting for publishers, because they will not get any additional readership for GPRC-labeled books.

For the case of Law specifically, it was mentioned that Law scholars publish more Dutch language publications. The interviewee from the discipline Law commented that the label is also being used for more professional publications. Within the discipline of Law, there exists a debate about the value and nature of research in Law and the place of doctrinal research. This debate has existed for some time (Stolker, 2003; Van Gestel and Micklitz, 2014; Kaltenbrunner and De Rijcke, 2017). Our respondent from the discipline Law had a nuanced view of the functionality of the GPRC label. With regards to the debate within Law on the distinction between scientific contributions and practice-oriented contributions, they felt that the GPRC label could offer a distinction between these different kinds of publications. However, the respondent added that it is relatively easy to get a GPRC-label for a publication, also for a more practice-oriented publication. This downplays the possibility that the GPRC label could be used to delineate between these different activities by Law researchers.

Over all, the three in-depth interviews provided a varied perspective on the GPRC label, with some points of difference. However, the respondents seemed to agree that the GPRC label is only a small element, and that its importance should not be overstated. The GPRC label was not seen as a label that is used by researchers when they select which scholarly books to consult by any of the respondents, and primarily seen as a way for authors to have their books be included in the VABB. The existing prestige associated with publishing at internationally renowned publishers remains intact. With regard to the professionalization of publishers, the interviewees painted a nuanced picture. On the one hand, publishers complied more with the requirements of the GPRC label after a few years. On the other hand, the respondents voiced doubts about the quality of the reviews, which may indicate that GPRC sometimes remains a formalistic exercise. An additional study could look into the peer review dossiers and also incorporate the position of the publishers to address this point.

## Discussion

In this segment, we attempt to provide explanations for the main trends found in the empirical part of the study and consequently discuss the implications of the GPRC label for bibliodiversity and balanced multilingualism. We also discuss the GPRC's potential to make the PRFS more inclusive toward different publication practices in SSH disciplines.

The GPRC label only accounts for a small proportion of total publications in the VABB. Moreover, none of the interviewees identified the GPRC label as an incentive toward publishing more at Flemish book publishers. Nonetheless, the GPRC label plays a part in a broader effort of recognizing the research activities SSH scholars in Flanders already perform and valuing them within the PRFS. As such, the label can protect part of the bibliodiversity of SSH in Flanders. In addition to opening an extra route for books as a typically less favored publication type, the label is open to local, typically small, publishers as well as publishers with a mixed portfolio.

From the point of view of the publishers, the GPRC label was meant as a way to redress the balance between publishers that are on the list of VABB-approved publishers and publishers that are not. However, the GPRC label is not always attractive to the publishers because of the added workload, the difficulty of finding reviewers and the limited benefits to the commercial attraction of their publications. This downplays somewhat the possibility of the label strengthening the market position of Flemish publishers.

The empirical results indicate that the label indeed caters mainly to Dutch-language publications. Moreover, the titles of History and Law publications with the GPRC label have shown that a focus on topics of local relevance exists among GPRC-labeled books. The empirical results have also shown a diversity in terms of the uptake of the label between the different disciplines, which points to a greater or smaller demand for a new channel for recognition of locally published scholarly books. Especially in Law, many publications in Dutch were added to the VABB through the GPRC label. These publications often discuss Belgian law in particular and are usually directed toward peers as well as professional law practitioners and students. A functional balance means that these types of publications exist alongside the English language publications in international outlets.

However, there are also potential drawbacks to Dutch language peer-reviewed books. Restricting peer reviewers to only colleagues from the same language area restricts the number of potential reviewers. Finding reviewers for scholarly books is important to a label for peer-reviewed books, and as the interviews have indicated, remains a concern. With the GPRC label, the burden of finding adequate reviewers lies with the publisher. Moreover, the GP does not evaluate the quality of the reviews. This is different from e.g., the Kriterium label in Sweden, where the reviewers are appointed by an academic coordinator, who in turn is appointed by the board governing the label (Hammarfelt et al., 2021). This exempts the publisher from the work associated with peer review and places the quality assurance in the hands of the label.

Apart from making the PRFS more inclusive toward regional publishers, the GPRC label also potentially alters the publication practices at those publishers. The increasing demand for peer review when evaluations focus more on the presence of peer review as a mark of quality create the necessity for these smaller publishers to adapt as well and instate peer review procedures where there were none before. The GPRC label offers a framework for having peer review at these publishers recognized as well as providing a formal set of rules peer-reviewed publications need to adhere to. This can help in a continuation of a bibliodiverse publishing landscape, but it also functions within a system where books need to be peer reviewed in order to “count.”

## Conclusion

Using a mixed approach consisting of data analysis, interviews and literature, we have discussed the GPRC label within its local context and analyzed its uptake and possible consequences. We show that the label provides an interesting solution to the problem of which books to include in local databases used for the allocation of research funding. The GPRC label is a flexible tool for recognizing individual book publications as peer-reviewed. In that way, it enables local publishers and researchers to have their publications included in the VABB more easily, without having to go through an appeals procedure. Furthermore, the GPRC label can contribute toward the goal to make the VABB database as comprehensive as possible. As such, total of 2,580 GPRC-labeled book publications have been included in the VABB in the period 2010–2019. Going forward, it seems to be important to continue to monitor the uptake of the GPRC label, and also to agree with both researchers and publishers on possible improvements to the functioning of the label. The data used in this study do not allow us to analyse what effect the GPRC label has had on the publishers and whether the publishers experience problems with the implementation of the label. We therefore suggest that future studies of the GPRC label take their perspective into account. Apart from that, an evaluation of the reviews submitted in the peer review dossiers could provide insight into the quality of the peer review. This last part, however, can only be attempted by enlisting independent reviewers from the different disciplines.

We have shown that the GPRC label is connected to bibliodiversity, in the sense that it allows books by smaller and medium-sized publishers in Flanders to be added to the VABB database. Aligning the PRFS with the specific publication practices of the SSH can be an element toward protecting the scholarly book publishing traditions of those disciplines. The results indicate that GPRC publications are often locally oriented, both in terms of topic (focus on Belgium or the Low Countries) and languages. The majority of publications were written in Dutch, which corroborates the idea that the GPRC label can contribute toward a “balanced multilingualism.” While the GPRC label will likely not convince authors to write their publications in Dutch, it will allow them to have their Dutch language publications published at local publishers to be counted in the PRFS.

We also found that the GPRC label was unevenly distributed among disciplines, with Law publishing by far the most GPRC-labeled book publications. The reason is that the different disciplines don't have the same use for the label. The label is most interesting for disciplines that publish locally oriented books at publishers that are not already included in the VABB. It certainly does not create a drive toward the publication of Dutch language books in those disciplines that have a predominantly journal-oriented international profile. This is not very surprising, but it does point toward the fact that while the GPRC label is part of a PRFS, it does not seem to have causal effects on the choice of publication outlet or language.

A final finding of this study concerns the aspect of peer review. The GPRC label has prompted publishers that previously did not have a formalized peer review procedure to adopt one in order to comply with the GPRC regulations. It remains to be seen whether publishers will continue to publish GPRC-labeled books, or whether some publishers may decide to publish fewer GPRC books because of the extra work involved in organizing the peer review procedure. Whereas the initiative for obtaining a GPRC label currently comes almost entirely from the author, publishers could become more interested in using the GPRC label if the label would be recognized within the scholarly community as a mark of quality. If the GPRC label would be reviewed, it would be helpful to take inspiration from other labels for peer-reviewed books that have recently been introduced. The Finnish and Swedish examples show that there are different uses of a label for books, the GPRC label's sole focus on approval in the VABB may be a limiting factor to its further uptake.

Finally, a few limitations of this study as well as suggestions for further research can also be given. Firstly, we have not taken book series into account in our analysis. Secondly, only limited attention was given to comparisons with the whole of the VABB. Thirdly, the total number of publications was quite small and the time frame limited to 10 years which makes it difficult to identify long-term trends. Another limitation is that we could not make causal claims about the effects of the GPRC label. Finally, future research could focus on analyzing in detail the developments at the Flemish publishers. Further studies could also explore the content of GPRC publications. While we offered a first look at the publication titles of Law and History publications, a closer analysis of both GPRC and non-GPRC publication titles and abstracts would enhance our understanding of the local and international characteristics of GPRC publications as well as the intended audiences of GPRC publications. This study also did not include an international comparison, in particular a comparison with the Finnish and Swedish labels for peer-reviewed publications could be interesting. Lastly, interviews with a larger number of authors from different disciplines as well as publishers of GPRC publications could give helpful suggestions for improving the label.

## Data Availability Statement

Part of the dataset is publicly available on: doi: https://doi.org/10.5281/zenodo.4472810. Part of the dataset is not available online. Requests to access these datasets should be directed to peter.aspeslagh@uantwerpen.be; raf.guns@uantwerpen.be; tim.engels@uantwerpen.be.

## Ethics Statement

The studies involving human participants were reviewed and approved by Ethics Committee for the Social Sciences and Humanities (EASHW) University of Antwerp. The patients/participants provided their written informed consent to participate in this study.

## Author Contributions

EV: conception and design, drafting the work, and performing the analysis. RG and TE: conception and design, substantial critical revisions, and providing approval for publication. All authors contributed to the article and approved the submitted version.

## Conflict of Interest

The authors declare that the research was conducted in the absence of any commercial or financial relationships that could be construed as a potential conflict of interest.

## Publisher's Note

All claims expressed in this article are solely those of the authors and do not necessarily represent those of their affiliated organizations, or those of the publisher, the editors and the reviewers. Any product that may be evaluated in this article, or claim that may be made by its manufacturer, is not guaranteed or endorsed by the publisher.
